# Dr. Wm. A. B. Norcom

**Published:** 1872-02

**Authors:** 


### DR. WM. A. B. NORCOM.

In this issue we publish the able an eminently practical address of the above named gentleman, read before the Medical Society of the State of North Carolina. This we have done, at the suggestion and request of one of our most accomplished Associates, of Virginia; and we feel assured that a careful perusal of this address will amply repay any of our medical friends for the time they may devote to that purpose. Dr. Norcom is a gentleman of fine culture and medical learning, and, without wishing to turn him away from his "first love," yet we would be pleased to hear from him, as a contributor, individually. He should not, we think, bury his fine talents, but write often for the medical press, for the benefit of his brethren. Among the number of journals opened to him, we trust he will not forget the solicitations of his friends and well-wishers of THE COMPANION.

